# Impact of the COVID-19 pandemic on Swedish adults aged 77 years and older: Age differences in lifestyle changes

**DOI:** 10.1177/14034948231172249

**Published:** 2023-05-07

**Authors:** Erika Augustsson, Isabelle Von Saenger, Neda Agahi, Ingemar Kåreholt, Malin Ericsson

**Affiliations:** 1 Aging Research Center, Karolinska Institute and Stockholm University, Sweden; 2 Aging Research Network, Jönköping University, Sweden

**Keywords:** Lifestyle factors, social contacts, COVID-19 pandemic, Sweden, older adults, Internet use, age

## Abstract

**Aims:**

This study aimed to describe the impact of the COVID-19 pandemic on lifestyle and social activities among older adults in Sweden, with a special focus on differences between the ‘younger old’ (aged 77–84) and ‘older old’ (aged 85–109).

**Methods:**

This study is based on a nationally representative sample of older adults (aged ⩾77 years) in Sweden (SWEOLD). Data were collected between May 2021 and April 2022, when many recommendations were removed but the virus was still classified as a public health disease. We studied occurrences and differences between the two age groups in several lifestyle factors and social activities.

**Results:**

The younger old displayed larger changes in lifestyles because of the pandemic than the older old. Most changes were found in social interactions with family.

**Conclusions:**

**Our results highlight the large heterogeneity within the Swedish population aged ⩾77 years, and that the younger old experienced a bigger lifestyle change than the older old. Previous activity levels might be important to consider in order to understand how regulations may affect the older population. Finally, our findings indicate large age differences in Internet use, which require attention to prevent digital exclusion of an already vulnerable group.**

## Introduction

The COVID-19 pandemic has caused suffering not only from death and disease but also from major changes to lifestyles and social life. In Sweden, the response to the pandemic differed from that of many other countries, for example there was no general lockdown. Apart from general regulations aimed at limiting the spread of the virus in society, there were also specific recommendations aimed at vulnerable populations. Based on the elevated mortality risk, those aged ⩾70 years was strongly urged to maintain social distancing, and nursing-home visits were banned during certain periods [1]. Recommendations did not consider the large diversity in those ⩾70 years of age regarding health, activity levels, family situation and independence, and thereby their levels of vulnerability.

Research has suggested that public health responses to the pandemic, such as lockdowns and social distancing, have impacted socio-demographic groups differently regarding social life and health behaviours [2]. Swedish studies have mostly focused on older urban populations during the pandemic’s first wave, showing that physical and social activities were largely reduced [3,4]. Internet use, particularly video calls, increased among older adults. However, the oldest old remain the least-common users [4,5].

We know little about lifestyle changes among older people in the entire Swedish population during the later parts of the pandemic. This article contributes to this knowledge gap by using a nationally representative sample of older Swedes (⩾77 years of age), including people living in institutions, when describing changes in lifestyle and social activities. This study has a special focus on differences between the ‘younger old’ (77–84 years of age) and the ‘older old’ (85–109 years of age). This can give us a better understanding of how older adults have adapted their lifestyles in response to the pandemic, and which lifestyle factors have seen the most change.

## Methods

This study is based on a nationally representative sample of those aged ⩾77 years in Sweden, using the 2021 wave of SWEOLD [6]. Respondents were interviewed by telephone and, as a second option, by postal questionnaires, including people living in institutions and proxy informants. The response rate was 63.6%. Data were collected from May 2021 to April 2022, when many recommendations were removed but the virus was still classified as a public health disease. For a description of the data, see www.sweold.se. The Swedish Ethical Review Authority Ethical approved the study (2021-00393).

The results are presented as proportions (adjusted for sex) standardised with marginal effects from OLS regressions. *p*-Values for age difference were based on logistic regression, with age group as the outcome.

Analyses were weighted using sample weights and conducted using STATA v17.0 (StataCorp, College Station, TX).

## Results

Compared to the general Swedish population aged ⩾77 years, our sample was representative in terms of sex, age and education (Table I). The older old (aged ⩾85 years) were more likely to be lower educated, live alone and live in institutions compared to the younger old (aged 77–84 years).

**Table I.** Descriptive statistics of the sample (%), not weighted.

	Official statistics^a^	Total *N*=848	Aged 77–84 years *N*=392	Aged ⩾85 years *N*=456
Sex				
Male	42.6	47.5	46.4	48.5
Female	57.4	52.5	53.6	51.5
Age (years)				
Mean (SD)	82.9 (5.1)	87.7 (8.1)	80.4 (2.2)	93.7 (6.1)
Range	77–100+	77–109	77–84	85–109
Education^7^				
Compulsory	34.5	34.5	34.5	41.9
Post-compulsory	63.8	65.5	65.5	58.1
Living alone		49.9	39.0	62.7
Institutional living	9.4^b^	16.9	3.1	28.7

a Data for the Swedish population aged 77+ on 31 December 2021 from Statistics Sweden.^8^

b Data are only available for those aged ⩾80 years.^9^

### Lifestyle factors

A vast majority (90%) perceived the COVID-19 recommendations as easy to understand, and 97% also followed them. Food and eating habits were not particularly affected by the pandemic. Life-space mobility was largely restricted, as many went outside or travelled less. While most reported no change, 19% reported less physical activity than before. These changes were more evident among the younger old than the older old (Table II).

**Table II.** Age-stratified descriptive statistics of self-reported changes in lifestyle factors related to the COVID-19 pandemic, adjusted for sex, % (CI).

	Total *N*=848	Aged 77–84 years *N*=392	Aged ⩾85 years *N*=456
COVID-19 recommendations			
Ease of understanding recommendations			
Very easy	32.3	32.8 (28.47, 37.06)	30.9 (23.71, 38.06)
Easy	58.1	56.8 (52.32, 61.37)	61.6 (54.08, 69.22)
Somewhat hard	6.7	7.6 (05.27, 09.84)	4.2 (00.37, 08.01)
Hard	3.0	2.8 (01.28, 04.39)	3.3 (00.68, 05.88)
Did not follow recommendations	3.3	3.0 (01.38, 04.70)	4.0 (01.23, 06.83)
Food and eating habits			
Eats cooked food daily	93.5	92.7 (90.71, 94.69)	95.4 (92.43, 98.46)
Change because of COVID-19	2.9	2.9 (01.51, 04.25)	3.1 (00.99, 05.15)
Does not buy food personally because of COVID-19	4.7	4.9 (03.10, 06.78)	4.0 (01.03, 06.96)
Life-space mobility			
Goes out/travels less since the start of the pandemic	40.3	41.3 (37.36, 45.33)	38.0 (31.95, 44.06)
Goes out/travels more since the start of the pandemic	3.3	3.4 (00.02, 00.05)	3.0 (00.01, 00.05)
Physical activity			
Daily	22.3	**25.6 (22.25, 28.95)**	**14.6 (09.47, 19.70)**
Several times a week	22.1	**26.2 (22.83, 29.49)**	**12.8 (07.73, 17.90)**
Once a week	6.4	7.1 (05.10, 09.07)	5.0 (01.92, 07.99)
1–3 times per month	2.5	2.3 (01.05, 03.57)	2.9 (00.97, 04.82)
Yes, but less often	5.7	6.3 (04.45, 08.22)	4.3 (01.40, 07.14)
No	40.9	**32.5 (28.66, 36.36)**	**60.5 (54.60, 66.36)**
Change in physical activity because of COVID-19			
Increase	4.7	4.7 (02.93, 06.37)	4.7 (02.02, 07.30)
Decrease	19.2	**21.3 (18.14, 24.54)**	**14.2 (09.29, 19.15)**
No change	76.1	**74.0 (70.54, 77.47)**	**81.1 (75.79, 86.46)**

Significance tests for age differences were performed using logistic regressions. Statistically significant age differences are presented in bold. Results are weighted using sample weights.

### Internet use and social contacts

More than half (60%) of the older old never used the Internet, whereas half of the younger old used it daily (Table III). The younger old used it more often (76% vs. 48%) to stay in touch with people. Internet use increased in both age groups.

**Table III.** Age-stratified descriptive statistics of self-reported changes in internet use and social interactions related to the COVID-19 pandemic, adjusted for sex, % (CI).

	Total *N*=848	Aged 77–84 years *N*=392	Aged ⩾85 years *N*=456
**Internet**			
Frequency of Internet use			
Daily	43.9	**51.7 (47.78, 55.58)**	**26.0 (20.13, 31.95)**
Several times a week	11.9	13.1 (10.44, 15.68)	9.3 (05.30, 13.28)
Approximately, one time a week	3.0	3.6 (02.22, 04.98)	1.6 (-00.53, 03.66)
1–3 times per month	1.4	1.8 (00.82, 02.75)	0.7 (-00.82, 02.12)
Seldom	2.1	2.0 (00.82, 03.17)	2.5 (00.70, 04.27)
Never	37.6	**27.9 (24.17, 31.60)**	**60.0 (54.33, 65.62)**
Uses Internet to communicate with people	70.6	**76.0 (71.25, 80.75)**	**47.9 (38.15, 57.67)**
Change in Internet use because of COVID-19			
Increase	28.0	29.1 (24.23, 33.94)	23.6 (13.54, 33.62)
Decrease	2.0	1.8 (00.26, 03.29)	3.0 (00.00, 06.17)
No change	70.0	69.1 (64.19, 74.09)	73.4 (63.15, 83.62)
No contact with relatives because of COVID-19^a^			
Not visiting relatives	17.4	**20.5 (17.47, 23.58)**	**10.1 (05.48, 14.77)**
Not having relatives over to visit	18.2	19.6 (16.45, 22.78)	14.9 (10.17, 19.68)
Not socialising with relatives outside the home	11.4	12.3 (09.73, 14.89)	2.0 (05.34, 13.21)
No contact with friends because of COVID-19^a^			
Not visiting friends	20.8	22.4 (19.15, 25.70)	16.9 (11.92, 21.88)
Not having friends over to visit	20.7	22.0 (18.69, 25.24)	17.7 (12.68, 22.64)
Not socialising with friends outside the home	14.3	15.7 (12.86, 18.54)	11.2 (06.87, 15.50)
Children			
Change in *in-person contact* with at least one child			
More	7.6	**5.8 (03.55, 08.10)**	**11.8 (08.31, 15.22)**
Less	61.0	**66.0 (61.89, 70.19)**	**49.4 (43.12, 55.73)**
Change in *phone/Internet contact* with at least one child			
More	20.3	21.6 (18.20, 25.10)	17.3 (12.04, 22.55)
Less	7.1	7.2 (04.95, 09.37)	7.0 (03.59, 10.32)
Grand and great-grandchildren			
Change in *in-person contact* with grand and great-grandchildren			
More	1.5	1.9 (00.83, 02.99)	0.5 (00.00, 02.19)
Less	52.0	54.5 (50.11, 58.99)	46.2 (39.50, 52.98)
Change in *phone/Internet contact* with grand and great-grandchildren			
More	6.6	7.3 (05.06, 09.46)	5.0 (01.66, 08.35)
Less	10.0	9.7 (07.07, 12.41)	10.7 (06.64, 14.77)

Significance tests for age differences were performed using logistic regressions. Statistically significant age differences are presented in bold. Results are weighted using sample weights.

a Question was: ‘Do you usually meet relatives/friends?’ Response option presented in table: ‘No, because of the pandemic’.

The younger old reported a greater decrease in social interactions with relatives and friends overall compared to the older old. There was an overall decrease of in-person contact with children and grandchildren, but for 20%, remote contact with at least one child increased. The younger old experienced a larger decrease of in-person contact with at least one child.

## Discussion

This study describes changes in lifestyles and social activities due to the COVID-19 pandemic in a nationally representative sample of Swedish older adults (aged ⩾77 years). Many people reported changes in lifestyle factors, especially in in-person contact with children and grandchildren, where the majority reported a decrease.

To highlight the heterogeneity of the older population, we explored differences between the younger old (aged 77–84 years) and the older old (aged ⩾85 years). Self-reported adherence to recommendations was similar across age groups, but the younger old made more lifestyle changes than the older old. Our findings suggest that the younger old were more active in terms of life-space mobility, physical activity and social contact with friends and relatives before the pandemic than the older old, resulting in a larger and presumably negative change.

Generally, levels of activities and social interactions tend to decrease with age [10], and restricting activities may therefore entail greater negative consequences for the younger old. In addition, this may have a negative impact on both mental and physical health [11,12]. These findings reflect the critique levelled at the governmental public health response regarding not taking the diversity of the older population into account.

We additionally explored Internet use and social contacts. Sweden is a highly digitalised country, where 94% of the population use the Internet daily [5]. However, this does not reflect the use in the older members of the population, as indicated by this study, where the proportion of users is considerably lower. Digital contact might partly compensate for lack of physical social contact [13], and while Internet use increased during the pandemic, there were large differences between the two age groups. More than 60% of the older old never used the Internet. This is worrying, indicating a digital divide and possible inequality regarding social and societal participation, especially in times of crises where important societal information is to be found online.

The data collection in this study was carried out during the end of the COVID-19 pandemic, but recommendations still applied. It is possible that the participants could have responded differently during an earlier part of the pandemic. This may have led to an under-reporting of negative impact. However, it allowed us to obtain a more comprehensive picture of the impact of the pandemic compared to studies that, for example, examined experiences during the first wave. Another strength of our data is the opportunity to make nationally representative age comparisons, as the data collection had no upper age limit and a relatively high response rate.

## Conclusions

Our results highlight the large diversity within the population aged ⩾77 years in terms of lifestyle behaviours and differences in how they have adapted their behaviours because of the pandemic. Older adults in Sweden have been responsive to the recommendations in place, and many have reduced their social interactions, which has disproportionately affected the younger old. Understanding age-group differences in lifestyle behaviours is important from a societal perspective, given that we have an increasingly older population. It is also important as a reference for times of crisis. The heterogeneity in lifestyles in such a large and diverse group of people should always be considered.
